# Signature Gene Mutations in Colorectal Cancer: Potential Neoantigens for Cancer Vaccines

**DOI:** 10.3390/ijms26104559

**Published:** 2025-05-09

**Authors:** Jaegoo Yoon, Haeun Moon, Yuna Jeon, Soohyun Choe, Hyunho Yoon

**Affiliations:** 1 Department of Medical and Biological Sciences, The Catholic University of Korea, Bucheon 14662, Republic of Korea; yoonjk999@catholic.ac.kr (J.Y.); ansgkdms1209@catholic.ac.kr (H.M.); dbsdk0620@catholic.ac.kr (Y.J.); cshyun16@catholic.ac.kr (S.C.); 2 Department of Biotechnology, The Catholic University of Korea, Bucheon 14662, Republic of Korea

**Keywords:** colorectal cancer, cold tumor, tumor microenvironment, signature genes, neoantigen, cancer vaccine

## Abstract

Colorectal cancer (CRC), the third most common cancer worldwide, is one of the deadliest cancers. CRC is known as a cold tumor, characterized by a low immune response that makes it difficult for immune cells to infiltrate and exhibits strong resistance to immunotherapy with checkpoint inhibition. This restricted response is largely attributed to signature gene mutations including mismatch repair (MMR) genes, *KRAS*, *BRAF*, *APC*, and *TP53*, which are also the main oncogenes in CRC. Mutated signature genes continuously upregulate abnormal signaling pathways, leading to excessive proliferation, cancer progression, and metastasis. Furthermore, it reorganizes the tumor microenvironment (TME) by recruiting immunosuppressive cells. However, the mutation can produce neoantigens that can provoke an immune response, making it a potential target for immunotherapy. In particular, cancer vaccines that leverage the strong neoantigenic properties of these mutations are considered promising for overcoming immune resistance and eliciting anti-tumor responses. In this review, we will describe signature gene mutations in CRC and focus on cancer vaccines targeting these mutations as potential therapies for CRC.

## 1. Introduction

Colorectal cancer (CRC) is one of the most deadly carcinomas, with 1.9 million incidence cases and 0.9 million deaths worldwide in 2020 [1]. Colorectal cancer can be classified based on the degree of microsatellite instability into three categories: microsatellite stable (MSS), microsatellite instability-high (MSI-H), and microsatellite instability-low (MSI-L) [2]. MSI-H is frequently caused by the loss of function of the DNA mismatch repair (MMR) genes, and the B-Raf proto-oncogene, serine/threonine kinase (*BRAF*) V600E gene mutation is also frequently observed [2]. While MSS CRC generally has normal DNA mismatch repair gene function, genetic mutations such as adenomatous polyposis coli (*APC*), Kirsten rat sarcoma viral oncogene homolog (*KRAS*), and tumor protein p53 (*TP53*) are commonly observed [3]. The genetic heterogeneity of CRC, driven by the accumulation of key gene mutations, regulates essential signaling pathways that play distinct roles in tumorigenesis [4,5]. Mismatch repair deficiency (MMR-D or dMMR) is a condition in which dysfunction in at least one of the four MMR genes impairs the recognition and repair of DNA mismatches during replication. This leads to increased mutagenesis in DNA and causes microsatellite instability (MSI) [6]. Mutations in the *KRAS* gene, present in approximately 40–50% of all CRC cases, lead to excessive activation of the RAS/RAF/MEK/ERK signaling pathway, promoting cell proliferation and inhibiting apoptosis [7]. Similarly, *BRAF* mutations also contributed to cell proliferation, differentiation, and survival through abnormal activation of the mitogen-activated protein kinase (MAPK) signaling pathway [8]. Somatic mutations in *APC* gene, a key regulator of the Wnt/β-catenin pathway, occur in approximately 70–80% of CRC cases and contribute to tumor progression [9,10]. Additionally, p53 mutations are observed in 43% of CRC cases, leading to the loss of wild-type p53 function and promoting tumor growth and metastasis [11]. Collectively, genetic mutations are associated with the formation of an immunosuppressive tumor microenvironment (TME), making them crucial targets for cancer therapy [12]. In recent years, CRC has been treated using various methods including immune checkpoint inhibitors (ICIs). However, CRC exhibited the characteristics of a cold tumor due to its immunosuppressive TME, limiting its ability to induce an effective immune response in patients [2,13].

TME consists of the surrounding environment of the tumor, including immune cells such as T and B lymphocytes, mesenchymal stromal cells, the extracellular matrix (ECM), and secreted molecules like growth factors, cytokines, and chemokines [14]. TME interacts with tumor cells to shape tumor heterogeneity. Depending on the activity of immune cells within the TME, tumor immunophenotypes are classified into two types: hot and cold [15,16]. A hot tumor, also known as an immune-inflamed tumor, exhibits a high immune response due to the presence of tumor-infiltrating lymphocytes (TILs). Conversely, a cold tumor, also referred to as an immune-excluded tumor, has few T lymphocytes, resulting in a low immune response [17]. In cold tumors, immunotherapy is less effective, necessitating the development of effective strategies to overcome the immunosuppressive [18]. Previous studies have shown that inhibiting dysregulated signaling pathways can relieve an immunosuppressive environment [19]. Therefore, new strategies targeting these mutations are needed to enhance the efficacy of CRC treatment.

Cancer vaccines utilize an ideal antigen that is highly immunogenic and exclusively expressed in cancer cells for immunotherapy [20]. Cancer vaccines employ neoantigens derived from tumor-associated antigens (TAAs) or tumor-specific antigens (TSAs) to induce robust activation of immune cells, including T lymphocytes, and to establish immunological memory by presenting antigens to antigen-presenting cells (APCs) [21,22,23]. In CRC, signature gene mutations have the potential to serve as neoantigens due to their tumor specificity and are emerging as promising targets for cancer vaccines [24,25]. Currently, in various clinical trials, cancer vaccines are improving patient prognosis while minimizing lesion development through the effective induction of an immune response [26]. However, cancer vaccines still face limitations in immunotherapy due to factors such as the heterogeneity of the TME, the presence of immunosuppressive cells, weak immunogenicity, and variations in patients’ immune systems depending on their human leukocyte antigen (HLA) type [27,28]. To overcome these limitations and improve the therapeutic efficacy of cancer vaccines, research targeting signature gene mutations is necessary and essential for further advancement.

This review aims to discuss the potential of signature gene mutations in CRC as promising targets for neoantigen-based cancer vaccines. Moreover, by presenting some cases of signature gene mutations targeted at cancer vaccines, we highlight the future prospects of cancer vaccine development.

## 2. Signaling Pathways and Characteristics of Signature Gene Mutations in CRC

**Figure 1:**
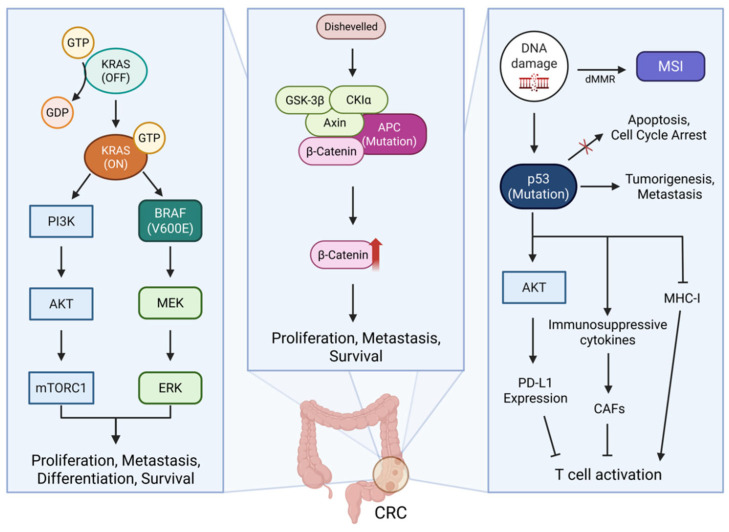
Signaling pathways of signature gene mutations in CRC. It illustrates the molecular interactions involving signature gene mutations, including MMR genes, *KRAS*, *BRAF*, *APC*, and *TP53*. Mutant *KRAS* continuously activate downstream signaling pathways such as PI3K, AKT, and mTORC1, thereby promoting CRC cell survival and proliferation. *BRAF* activates MEK and ERK driving tumor cell proliferation and immune modulation. Abnormal APC fails to properly degrade β-catenin, leading to its accumulation. This activates the transcription of Wnt target genes involved in cell proliferation, survival, and metastasis, thereby promoting an immunosuppressive TME. Mutant p53 also facilitates cell proliferation and deregulated cell cycle progression. Moreover, they downregulate MHC class I expression, increase immunosuppressive cytokine production, and enhance immune evasion by upregulating PD-L1 and stimulating CAFs. CRC, colorectal cancer; CAFs, cancer-associated fibroblasts; MHC, major histocompatibility complex; PD-L1, programmed cell death-ligand1; dMMR, mismatch repair deficiency; GSK-3β, glycogen synthase kinase-3β; CK I α, casein kinase I alpha; APC, adenomatous polyposis coli; MSI, microsatellite instability; KRAS, Kirsten rat sarcoma virus; PI3K, phosphatidylinositol 3-kinase; AKT, protein kinase B; mTORC1, mammalian target of rapamycin complex1; MEK, mitogen-activated protein kinase; ERK, extracellular signal-regulated kinase.

The signature gene mutations (MMR, *KRAS*, *BRAF*, *APC*, and *TP53*) in CRC are well known for playing significant roles in proliferation, invasion, migration, and metastasis [29]. The signaling pathways mediated by signature gene mutations are intricately interconnected, promoting CRC progression [30]. However, from an immunotherapeutic perspective, the mutated sequences of specific genes can serve as promising targets for cancer vaccines by providing neoantigens for effective anti-cancer strategies [31]. In this section, we highlight the signaling cascades of signature genes in CRC (Figure 1).

### 2.1. MMR in CRC

The MMR system is highly conserved and functions to excise base-base mismatch, thereby correcting errors that occur during DNA replication [32,33]. Its role is closely linked to the DNA damage response, which activates cell cycle arrest and apoptosis to prevent tumorigenesis caused by DNA mismatches [33]. In other words, MMR genes are crucial for maintaining the fidelity of DNA replication, so the inactivation of MMR genes inevitably leads to the spontaneous acquisition of a mutated phenotype in normal cells [33]. Mutations in MMR genes cause frequent somatic mutations, particularly in microsatellite regions, leading to MSI, which is associated with a high tumor mutation burden (TMB). In contrast, MSS exhibits the opposite characteristics [6]. MSI is associated with mutations in several MMR genes, including mutL homolog1 (*MLH1*), must homolog2 (*MSH2*), must homolog6 (*MSH6*), postmeiotic segregation increased2 (*PMS2*), or in the epithelial cell adhesion molecule (EPCAM) [32,34]. MSI CRC is characterized by hypermethylation of the *MLH1* promoter, leading to its inactivation, and is frequently accompanied by the *BRAF* V600E mutation [35]. While MMR and MSI are not synonymous, their close interconnection carries important diagnostic and clinical implications. MMR gene analysis can be used as a diagnostic approach for evaluating MSI [36]. Unlike the MSS CRC, which exhibits low levels of TILs, MSI-H CRC is highly infiltrated with CD8+ and CD4+ T cells. Therefore, immune checkpoint inhibitors including nivolumab and ipilimumab can be used to treat MSI CRC and have shown effective responses [37]. Although they exhibit positive responses to ICIs, immune evasion mechanisms—such as the upregulation of Wnt/β-catenin signaling, Foxp3⁺ regulatory T cells (Tregs), and immune checkpoint molecules—can attenuate or interfere with their therapeutic efficacy [38]. Recently, to enhance the efficacy of ICIs for MSI-H CRC, combination therapy with cancer vaccines targeting shared neoantigens has been shown to be safe and to exhibit strong anti-tumor effects [34].

### 2.2. KRAS in CRC

Mutations in the RAS family of genes are frequently found in various malignancies, including CRC. RAS proteins act as key regulators of tumor cell proliferation, migration, and differentiation [39]. The *RAS* gene encodes three homologous RAS proteins: HRAS, NRAS, and KRAS [40]. In CRC, *KRAS* mutations are detected in more than 40% of cases [41]. These mutations, which commonly occur at codons 12, 13, and 61, lead to amino acid substitutions that result in structurally altered KRAS proteins, with codon 12 mutations being the most prevalent [42]. G12D (glycine 12 to aspartic acid) and G12V (glycine 12 to valine) are the most common subtypes of *KRAS* mutations in CRC, due to their low GTP hydrolysis rate compared to other mutations. In particular, G12D mutations occur in approximately 50% of cases [40,43]. *KRAS* mutations are influenced by the protein’s ability to hydrolyze GTP, and when nucleotide substitution is enhanced, downstream signaling pathways become activated [7]. Normally, KRAS toggles between ON and OFF states through its intrinsic GTPase activity. When GDP is exchanged for GTP, KRAS switches to the ON state and activates the MAPK/PI3K pathway [41]. The MAPK pathway involves sequential phosphorylation of downstream kinases—RAF, MEK, and ERK—which activate transcription factors, leading to cellular proliferation, migration and survival [44]. Mutant KRAS proteins with defective GTPase activity remain locked in the “ON” state, persistently activating downstream signaling and driving uncontrolled cell proliferation, a hallmark of CRC [42,45]. Also, mutations in the *KRAS* gene enhance cancer cell adaptation to metabolic stress. Mutant *KRAS* allows cancer cells to survive and proliferate under glucose-deprived conditions, unlike normal cells or *KRAS* wild-type cancer cells [46].

*KRAS* has long been regarded as an undruggable target due to its structural and biochemical properties. However, recent studies have shown that various immunotherapeutic approaches exhibit promising efficacy in targeting *KRAS*-driven cancers [47]. Notably, it has been suggested that combining immunotherapy with allele-specific KRAS inhibitors can improve anti-tumor efficacy in *KRAS* mutant cancers [48]. Other promising immunotherapy strategies, including adoptive T cell transfer targeting mutant *KRAS* and *KRAS*-targeted mRNA vaccines, are designed to stimulate tumor-specific T cell responses [49]. The structural changes caused by these mutations lead to the formation of unique mutant peptides not present in normal tissues, making them an attractive therapeutic target [50]. These mutant peptides can be presented on the surface of tumor cells by major histocompatibility complex (MHC) molecules, allowing their recognition by T cells as neoantigens [51]. Unlike TAAs, which may also be expressed in normal tissues, *KRAS*-derived neoantigens are tumor-specific, reducing the risk of off-target effects and making them highly attractive candidates for cancer immunotherapy [52]. Studies reporting rapid tumor regression in CRC patients treated with *KRAS* G12D-specific T cells highlight the potential of *KRAS* mutations as promising neoantigen targets for immunotherapy [2].

### 2.3. BRAF in CRC

BRAF is a serine/threonine kinase that plays a critical role in the MAPK signaling pathway alongside KRAS, regulating various cellular processes such as growth, differentiation, and survival [53]. As a member of the RAF kinase family, BRAF functions downstream of KRAS, where it activates MEK1/2, which in turn activates ERK1/2 to transduce cellular signals [54]. Interestingly, *BRAF* and *KRAS* mutations are mutually exclusive, meaning that they typically do not occur together in the same tumor [55]. While both mutations result in activation of the MAPK pathway, they operate through distinct mechanisms. BRAF kinase functions independently of RAS-GTP binding and RAF dimerization, leading to continuous activation of the signaling pathway in the presence of specific mutations [56]. Wild-type BRAF forms a negative feedback loop upstream in response to excessive ERK activation. In contrast, hyperactivation of the MAPK pathway due to genetic alterations contributes to CRC progression [57]. In CRC, 8–12% of patients with BRAF alterations carry the V600E mutation, which involves a substitution of valine (V) with glutamic acid (E) at position 600 in the BRAF protein [55,58]. The V600E mutation causes BRAF to become autonomously active by bypassing the normal regulatory mechanisms that control its activation [59]. This persistent activation of the MAPK signaling pathway leads to uncontrolled cell proliferation, resistance to apoptosis, and tumor progression—all of which are hallmarks of malignancy [53]. Additionally, the influence of *BRAF* mutations extends to the immune environment in CRC. The mutation not only drives tumor cell proliferation but also induces immune modulation, contributing to an immune evasion phenotype [60]. In MSI-H CRC, it can lead to an upregulation of immune checkpoint proteins, such as programmed cell death ligand 1 (PD-L1), which inhibits T cell-mediated immune responses. Although the altered immune environment is characterized by increased infiltration of neutrophils and M1 macrophages, reducing the presence of naïve CD4+ T cells, resting dendritic cells, and plasma cells are typically involved in immune tolerance [61]. These immune changes make *BRAF*-mutant CRC particularly challenging to treat, as the immunosuppressive TME hinders the effectiveness of many therapeutic strategies.

Recent studies have revealed that targeting the *BRAF* V600E mutation shows promise in clinical settings. Agents such as vemurafenib and encorafenib have demonstrated effectiveness in selectively inhibiting mutant *BRAF*, leading to tumor shrinkage in some cases of CRC [62,63]. However, even when BRAF is suppressed, the MAPK pathway is often reactivated through bypass mechanisms, necessitating combination therapy to overcome resistance. Ex vivo studies of T lymphocytes targeting mutant forms of *BRAF* or associated *RAS* mutations have also shown significant results [64]. This approach not only allows for the direct targeting of tumor cells but also modulates the immune TME, supporting other therapeutic strategies for anti-tumor activity [55]. Another strategy involves using TAAs to stimulate T cell responses through vaccination, offering hope for future therapeutic paradigms [65].

### 2.4. APC in CRC

The *APC* gene is a tumor suppressor gene that encodes a multidomain protein. *APC* plays a crucial role in regulating various cellular processes through interactions with multiple binding partners. These processes include chromosome segregation, cell migration, apoptosis, adhesion, proliferation, and differentiation. The diverse functions of *APC* highlight its significance in maintaining cellular homeostasis and preventing tumorigenesis [66]. Moreover, *APC* plays a crucial role in the activity of the Wnt/β-catenin signaling complex, interacting with components such as GSK3β, Disheveled, and Axin, and functioning as a key regulator of the Wnt signaling pathway. It also modulates the cell cycle and maintains chromosomal stability [67]. In the Wnt/β-catenin signaling pathway, which regulates at least 80 target genes, normal expression of the *APC* gene ensures proper degradation of β-catenin through phosphorylation and ubiquitination, thereby suppressing tumor formation. Maintaining low levels of β-catenin in the nucleus is important for the negative feedback regulation within the Wnt/β-catenin pathway. However, when APC functions abnormally, the accumulation of β-catenin triggers the transcription of Wnt target genes associated with cancer cell proliferation, survival, and metastasis, thereby creating an immunosuppressive TME [68,69,70]. It has been revealed that *APC*-mutated CRC exhibits elevated expression levels of vascular endothelial growth factor A (VEGFA) and marker of proliferation Ki67 (MKI67)along with reduced proportions of CD3⁺ and CD68⁺ cells. These findings indicate that *APC* mutations are associated with tumor invasion and proliferation [71]. In the early stages of CRC development, approximately 80% of CRC harbor inactivating mutations in the *APC* gene. *APC* mutations are prevalent across all stages of CRC progression [72]. These mutations initiate 80–85% of sporadic CRC cases, with the exception of MSI CRC status, which results from MMR deficiency [73]. Given the high prevalence of *APC* gene mutations, they hold potential as predictive biomarkers in CRC.

### 2.5. TP53 in CRC

The *TP53* gene, a tumor suppressor gene, plays a critical role in preventing cancer development. It encodes the p53 protein, a transcription factor that regulates the cell cycle, DNA repair, senescence, and apoptosis, thereby preventing abnormal cell proliferation and division [66,74]. Wild-type p53 functions as a direct activator of the *WAD-1* gene, which is induced to suppress tumor cells within the signaling pathway [66]. Programmed cell death pathways (PCD) are essential for the body to maintain homeostasis. *TP53* is involved in regulating various types of PCD pathways, including apoptosis, autophagy, pyroptosis, and ferroptosis, as well as pathways related to reactive oxygen species (ROS) generation [74]. Under normal conditions, the p53 protein is continuously degraded by MDM2, maintaining low intracellular levels. However, when cellular stress occurs, such as DNA damage due to environmental stimuli or spontaneous replication errors, or when oncogenes and ROS are activated, p53 degradation by MDM2 is inhibited to maintain homeostasis. As a result, p53 protein accumulates, leading to elevated p53 levels [75,76]. As p53 levels increase, it promotes cell death by upregulating apoptosis-related proteins such as PUMA and NOXA [74,77].

The *TP53* gene is one of the most frequently mutated genes in human malignancies. *TP53* mutations are commonly observed in CRC [75,78]. *TP53* mutations contribute to the progression of adenomas into malignant tumors in colorectal cancer by impairing apoptotic pathways, thereby promoting cellular proliferation and uncontrolled cell cycle progression [66]. More than 20% of *TP53*-mutated CRC harbor missense mutations at positions R175 or R273. These two mutational hotspots are known to promote tumor metastasis and reduce survival rates in CRC, suggesting that these specific *TP53* mutations may play a significant role in CRC progression and patient outcomes [79]. The R175H mutation induces protein misfolding, resulting in structural alterations in the p53 DNA-binding domain. *TP53* mutations harboring the R175H mutation enhance tumorigenic potential and increase the expression of drug resistance genes, leading to a poor prognosis for CRC patients [78,80]. Additionally, CRC tumors with R273 mutations are more likely to progress to metastatic diseases which are associated with lower survival rates. The R273H mutation regulates a specific transcriptional program that activates oncogenic signaling pathways and promotes the development of more aggressive tumors [79,81]. *TP53* mutations, including those at the R273 position, decrease MHC-I expression and increase the secretion of immunosuppressive cytokines, thereby promoting the activation of CAFs. The activation of CAFs leads to the creation of a cold tumor environment, which acts as a mechanism to enhance immune evasion. Furthermore, overexpression of *TP53* mutations increases PD-L1 expression levels by activating AKT, thereby inhibiting T cell activation [82]. Notably, when accompanied by *KRAS* mutations, a more potent immunosuppressive TME is established, ultimately resulting in immune escape [82,83].

## 3. Immunological Function of TME in CRC

CRC is a malignancy characterized by a complex TME, in which cellular and non-cellular components interact to facilitate immune evasion, tumor progression, and therapeutic resistance [84]. Cellular components (e.g., endothelial cells, CAFs, T and B lymphocytes, APCs, and mast cells) and non-cellular components, such as ECM, cytokines, growth factors, and vascular structures, are intricately interconnected, contributing to immunosuppression [85,86,87]. Furthermore, cancer cells actively remodel the TME by recruiting immunosuppressive mediators such as myeloid-derived suppressor cells (MDSCs), Tregs, tumor-associated macrophages (TAMs), and CAFs, while simultaneously modulating their own immunogenicity through signature gene mutations [88,89]. For these reasons, CRC is classified as a “cold tumor,” exhibiting poor responsiveness to immunotherapy [90]. As cold tumors, TME in CRC suppresses infiltrating T cells, NK cells, and dendritic cells, leading to a non-inflamed phenotype [17,83,91].

**Figure 2:**
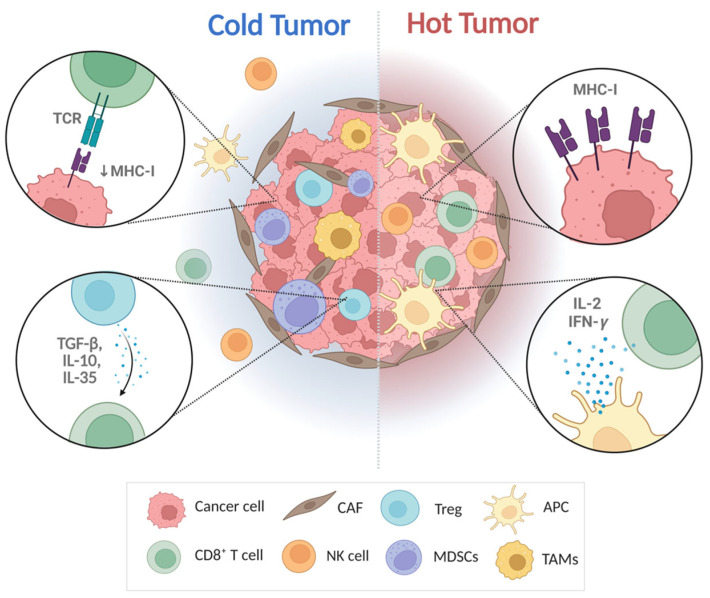
Differences in immune microenvironment between “cold and hot” tumors in CRC. The TME is composed of a complex structure with tightly interconnected cellular components—such as CAFs, Tregs, APCs, NK cells, and MDSCs—and non-cellular components. Hot tumors are characterized by high levels of infiltration by CD8+ T cells, NK cells, and APCs, resulting in an inflammatory phenotype. They secrete pro-inflammatory cytokines, such as IL-2, IFN-γ, and cancer cells present relatively high MHC-I. In contrast, cold tumors include immunosuppressive mediators, such as MDSCs, Tregs, TAMs, and CAFs, leading to a non-inflammatory phenotype. They suppress presenting MHC-I, and secrete anti-inflammatory cytokines, such as TGF-β, IL-10, and IL-35. TCR, T cell receptor; MHC, major histocompatibility complex; TGF, transforming growth factor; IL, interleukin; IFN, interferon; CAF, cancer-associated fibroblast; Treg, regulatory T cell; APC, antigen-presenting cell; NK cell, natural killer cell; MDSC, myeloid-derived suppressor cell; TAMs, tumor-associated macrophage.

MDSCs secrete various molecules, including nitric oxide (NO), ROS, TGF-β, and cytokines such as IL-1 and IL-6 [83]. These molecules promote an acidic environment through lactic acid–induced hypoxia-inducible factor-1 alpha (HIF-1α), leading to the expression of immune checkpoint molecules such as PD-L1 [92,93]. In particular, the accumulation of ROS and NO promotes tumorigenesis while suppressing anti-tumor immune responses [93,94]. TAMs also contribute to immune evasion under hypoxic conditions alongside MDSCs [17]. TAMs, in cooperation with Tregs, transform the TME into an anti-inflammatory state by secreting chemokines such as CC Motif Chemokine Ligand 2 (CCL2), CC Motif Chemokine Ligand 5 (CCL5), VEGF, and Transforming growth factor beta (TGF-β), thereby promoting the growth and progression of advanced CRC [95]. Specifically, *KRAS* mutations influence the polarization of macrophages toward a TAM-like phenotype, characterized by increased expression of immunosuppressive cytokines such as IL-10 and TGF-β [96]. Tregs secrete TGF-β, IL-10, and IL-35, which induce T cell exhaustion and suppress antigen presentation by APCs [97]. In cancers harboring both *KRAS* and *TP53* mutations, an increased infiltration of Tregs has been observed [98]. CAFs, as a key component of the tumor stroma responsible for synthesizing and maintaining the ECM, play critical roles in tumor initiation and progression [99]. CAFs can be broadly classified into two subtypes: inflammatory CAFs (iCAFs) and myofibroblastic CAFs (myoCAFs). iCAFs are characterized by elevated secretion of IL-6, whereas myoCAFs predominantly secrete TGF-β. Notably, CRCs primarily exhibit the iCAF phenotype, leading to increased IL-6 production and enhanced MDSC recruitment. This process is further exacerbated by *KRAS* mutations, which promote CXCL1 transcription and enhance MDSC mobilization into the tumor microenvironment [100,101]. These mechanisms provide a foundation for cancer cells to evade immune surveillance and develop resistance to immunotherapy. Comprehensively, converting cold tumors into hot tumors is a central strategy in cancer immunotherapy, as it can enhance the effectiveness and response rate of treatment (Figure 2).

## 4. Applications of Cancer Vaccines Targeting Signature Gene Mutations in CRC

As mentioned above, CRC is characterized by frequent mutations in specific genes, including MMR, *KRAS*, *BRAF*, *APC*, and *TP53*, which contribute to tumor progression and immune evasion. Given their crucial roles in shaping the TME, these signature gene mutations are regarded as promising targets for immunotherapy. Cancer vaccines offer an alternative immunotherapeutic approach by priming and expanding tumor-specific T cells before tumor-mediated immune suppression becomes dominant. For this reason, cancer vaccines have emerged as a promising strategy by inducing tumor-specific immune responses and providing a potential approach for CRC treatment [20,102]. Moreover, cancer vaccines can be combined with ICIs to enhance their efficacy by increasing T-cell infiltration in MSS CRC, thereby overcoming one of the major resistance mechanisms to ICI therapy [103]. The main mechanism of cancer vaccines is to introduce selected antigens into APCs to elicit immune responses [21]. Cancer vaccines can be classified into cell-based, peptide-based, nucleic acid-based, and viral vector-based types, depending on the method of APC activation.

### 4.1. Types of Cancer Vaccines

#### 4.1.1. Cell-Based Vaccine

DCs, as key antigen-presenting cells in peripheral tissues, are responsible for capturing, processing, and presenting antigenic peptides, including TAAs and TSAs. They are pivotal in initiating and regulating CD8⁺ and CD4⁺ T cell responses, particularly in antitumor immunity [104]. Cell-based cancer vaccines, particularly DC vaccines, harness a patient’s dendritic cells to elicit specific immune responses that selectively eliminate target cells. These cells are activated ex vivo by loading them with autologous antigens, after which they are reintroduced into the body [105,106]. Another approach to cellular vaccination involves using whole-cell preparations derived from tumor cells. These cells are either inactivated or genetically modified to prevent pathogenicity. They are recognized by various immune cells, including dendritic cells, macrophages, and NK cells, thereby eliciting broad, non-specific immune responses [27]. These vaccines can serve as safe adjuvants to enhance the efficacy of other anticancer therapies, such as chemotherapy and ICIs [107]. A representative DC-based cancer vaccine is Provenge (sipuleucel-T), the first FDA-approved therapeutic cancer vaccine for prostate cancer [108]. In CRC, GVAX, composed of CRC cells and used in an adjuvant setting, has demonstrated safety and the ability to enhance antitumor immunity [109]. However, a major limitation of cell-based vaccines is the potential for HLA mismatch, which can compromise vaccine efficacy. Therefore, the use of personalized elements such as neoantigens is essential to maximize therapeutic effectiveness.

#### 4.1.2. Peptide-Based Vaccine

Peptide-based cancer vaccines use synthetic peptides to stimulate the immune system. These peptides typically consist of short amino acid sequences containing specific antigenic epitopes [110]. Peptide length influences the type of immune response elicited. Short peptides (8–11 amino acids) are presented by MHC-I molecules and recognized by CD8⁺ T cells, leading to the generation of cytotoxic T lymphocytes (CTLs). In contrast, long peptides (11–30 amino acids) are presented by MHC-II molecules and recognized by CD4⁺ T cells, inducing helper T cell responses [111,112]. The selection of appropriate tumor antigens is critical in designing peptide vaccines. Targeting neoantigens—abnormally overexpressed or mutated proteins specific to cancer cells—is essential for effective cancer vaccine development [112]. Peptide-based vaccines alone are often insufficient due to their low immunogenicity. Therefore, potent adjuvants or immune stimulants are required to induce robust immune responses [113]. Despite certain limitations, peptide-based vaccines remain an attractive therapeutic option due to their ease of synthesis and low production cost [111]. Peptide-based vaccines are currently being evaluated in clinical trials, and in CRC, ELI-002 targeting *KRAS* mutations has demonstrated safety and promising therapeutic efficacy [114,115]. NCT04117087 is a Phase I study investigating a synthetic long peptide (SLP) KRAS vaccine in combination with dual checkpoint blockade (ipilimumab and nivolumab). The trial aims to enhance immune responses in cold tumors, including MSS CRC. Overall, induction of KRAS-specific T cell responses has been associated with improved survival [116,117]. These findings support peptide-based cancer vaccines as a promising strategy for CRC treatment.

#### 4.1.3. Nucleic Acid-Based Vaccine

Nucleic acid-based cancer vaccines deliver plasmid DNA (pDNA) or mRNA into APCs to activate the immune system [118]. Nucleic acid-based vaccines offer unique advantages, including rapid development, efficient manufacturing, and ease of customization [119]. Furthermore, nucleic acid vaccines have demonstrated favorable safety profiles compared to traditional vaccines and viral vector platforms, which rely on live-attenuated or replication-deficient viruses. Briefly, nucleic acid-based vaccines are more efficient, easier to produce, and readily modifiable than other vaccine types [120]. mRNA vaccines have emerged as a promising therapeutic platform since the COVID-19 pandemic. They allow flexible sequence design and enable the encoding of tumor antigens, thereby inducing both innate and adaptive immune responses. Additionally, unlike DNA vaccines, mRNA vaccines eliminate the risk of host genome integration [120]. Due to the instability of mRNA vaccines, an optimized delivery system is essential for protection against degradation, with lipid nanoparticles (LNPs) serving as a key formulation for effective mRNA delivery [121]. Despite remaining challenges such as potential side effects and limited efficacy, various mRNA-based cancer vaccines are currently undergoing clinical trials [119,122]. BNT122 (NCT04486378), an mRNA-based vaccine, is being investigated for the treatment of stage II/III CRC. This study demonstrates that BNT122 induces neoantigen-specific T cell responses, leading to prolonged disease-free survival [123]. In addition, mRNA-5671 (NCT03948763), targeting *KRAS* mutations, is undergoing a Phase I trial in combination with pembrolizumab, a PD-1 inhibitor [119]. These studies underscore the growing potential of mRNA-based vaccines as a novel approach to cancer immunotherapy.

#### 4.1.4. Viral Vector-Based Vaccine

Viruses inherently possess immunogenic properties, and their genomes can be engineered to deliver transgenes for expression in host cells, including immune cells [124]. Therefore, they can elicit strong and long-lasting immune responses without the need for adjuvants. Various viruses are utilized as recombinant viral vectors, including adenoviruses, poxviruses, herpesviruses, and lentiviruses [125]. These viral vectors are human-compatible, non-immunogenic, and non-pathogenic, and offer efficient gene delivery compared to other vaccine platforms [126,127]. While conventional viral vector vaccines aim to stimulate immune responses via APCs, oncolytic virus therapy represents an alternative viral approach that directly targets and destroys cancer cells [127]. It aims to identify, infect, and lyse cancer cells or cells within the TME to inhibit tumor progression and activate antitumor immune responses [128]. Nevertheless, oncolytic viruses are potentially strong therapies for cancer treatment, they cannot be enough to eliminate all the cancer cells because of the heterogeneity and complicated structure of tumors [129]. Thus, further research is required to advance both viral vector-based vaccines and oncolytic virus therapies. The first approved oncolytic virus drug, T-VEC, is based on a genetically modified herpes simplex virus type 1 encoding granulocyte-macrophage colony-stimulating factor (GM-CSF) and is used to treat unresectable melanoma [130]. In CRC, Pexa-Vec, an oncolytic virus therapy, is undergoing Phase I/II clinical trials in combination with ICIs. This combination has shown promising activity in advanced MSS CRC [131,132]. NCT03953235 is a viral vector-based vaccine composed of chimpanzee adenovirus (ChAd68) and self-amplifying mRNA (samRNA). This shared neoantigen vaccine, when combined with ICIs, has been shown to extend median progression-free survival and overall survival [133]. Consequently, ongoing research on both viral vector vaccines and oncolytic virus therapies is essential to address current challenges in cancer treatment.

### 4.2. Advantages and Challenges of Cancer Vaccines Targeting Signature Genes

Various cancer vaccines targeting signature mutations in CRC are being developed using diverse platforms each with distinct mechanisms (Table 1). In this section, we summarize vaccine candidates currently being studied and discuss their advantages and strategies to overcome challenges. GVAX, cell-based vaccines have been designed to stimulate broad immune activation by secreting GM-CSF [134]. Despite eliciting strong immune responses, their efficacy as monotherapies has been constrained by the immunosuppressive TME and insufficient antigen specificity. Similarly, TG01, a peptide vaccine designed to induce DTH and T-cell proliferation, has achieved immune activation but only modest clinical benefits when used alone [135]. These suggest that combination strategies, such as pairing with ICIs or T-cell modulators, may be critical to fully amplify the therapeutic potential of these vaccines. To enhance immune responses, some platforms focused on boosting antigen-specific T-cell activation. ELI-002, a lipopeptide-based vaccine utilizing AMP-CpG technology, has demonstrated that [136], although sustaining durable memory responses remains a challenge. Long peptide-based vaccines targeting KRAS neoantigens have shown the ability to broaden T-cell diversity by activating both CD4⁺ and CD8⁺ subsets. Ongoing efforts to optimize antigen selection, epitope processing, and delivery methods are expected to further improve the clinical efficacy of these peptide-based platforms. E-39 has shown immune engagement via cytolysis with TALs [137], and thus enhancing the persistence and functionality of activated T cells may strengthen the durable therapeutic outcomes. More recent advancements have aimed to fundamentally address tumor heterogeneity and recurrence at their origin. The KISIMA-Ascl2 vaccine, targets ISC-related antigens, and is promising to combat tumor stemness and therapy resistance [138]. This approach holds significant potential for achieving long-term disease control even though it is still in the early clinical stages. Moreover, mRNA-based vaccines such as BNT122 and mRNA-5681 have emerged as highly adaptable tools for inducing tumor-specific cytotoxic T-cell responses and antigen expression [139,140]. Vector-based strategies, GRT-R904 employs a prime-boost approach [133], and oncolytic virus-based vaccines like Pexa-Vec (JX-594) directly lyse tumor cells and stimulate systemic immunity [131], further diversify the vaccine landscape. Nevertheless, overcoming hurdles such as vector-specific immune clearance and sustaining robust immune memory remains essential to promote their effectiveness. Finally, Nous-209 offers an alternative strategy by inducing FSP-specific T-cell responses promise in MSI-H CRC [141], although broader validation in MSS CRC remains an important next step. Taken together, these show significant progress in CRC immunotherapy, each addressing unique aspects of tumor immunity. To maximize their therapeutic potential, refining antigen targeting, immune activation, and combination strategies is pivotal. With ongoing study and clinical validation, these vaccines offer strong potential for durable, effective treatments against colorectal cancer.

### 4.3. Development and Limitations of Signature Gene-Targeted Vaccines in CRC

*KRAS* and *BRAF* mutations have remained the primary focus of vaccine development in clinical trials, as noted above [145]. These mutations generate strong tumor-specific neoantigens, making them attractive targets for cancer vaccine development. In particular, *KRAS* mutations such as G12D, G12V, and G13D produce neoantigens recognizable by the immune system, eliciting CD8⁺ T cell responses. Similarly, the *BRAF* V600E mutation generates a well-characterized neoantigen that has been actively explored in immunotherapy strategies [48,146]. *TP53* mutations are primarily associated with loss-of-function, which means that they fail to produce stable and immunogenic neoantigens suitable for vaccine targeting; however, certain gain-of-function phenotypes can elicit robust cytotoxic and T helper cell-mediated immune responses [31]. Simultaneously targeting *KRAS* and *TP53* mutations using long-peptide vaccination induced strong cytotoxic and helper T cell responses [147]. In addition, various vaccine platforms have been employed to target mutant *TP53*, aiming to enhance immune responses by increasing CD8⁺ T cell activity [148]. Since mutant p53-derived peptide neoantigens are commonly present in various cancer types, they represent a promising target for cancer vaccine development [149]. *APC* mutations have been proposed as potential sources of tumor-specific neoantigens for cancer vaccine development [150]. However, a major challenge is that *APC* mutations primarily result in loss-of-function, which limits their capacity to generate directly immunogenic neoantigens [151,152,153]. Consequently, recent research has expanded the concept of neoantigens beyond peptides derived solely from gain-of-function mutations [154]. Loss-of-function mutations may indirectly give rise to neoantigens by causing the aberrant expression of associated signaling proteins. This shift in perspective has led to the identification of overexpressed signaling components as promising vaccine targets in CRC [155]. A recent study has evaluated the efficacy of the KISIMA-Ascl2 vaccine, which targets the self-antigen Ascl2, in an *APC*-mutant mouse model (Apc^+/Min-FCCC^ mice) [138]. Ascl2 is a key regulator in the Wnt/β-catenin pathway and is overexpressed due to *APC* mutations [156]. Following vaccination, both T-cell and antibody responses were significantly enhanced in mice. Notably, the immune response was further amplified and exhibited a stronger tumor-suppressive effect when combined with anti-PD-1 antibodies. These results demonstrated that the KISIMA-Ascl2 vaccine effectively suppressed colonic adenoma formation induced by *APC* mutations, while also confirming its immunogenicity and safety [138]. Similarly, MMR gene mutations are difficult to directly target with vaccines; however, they can generate frameshift-derived neoantigens that are targetable [157]. Current research on vaccines for dMMR CRC primarily focuses on utilizing these neoantigens. For instance, Nous-209, a vaccine targeting 209 frameshift peptides derived from dMMR/MSI-H tumors, has demonstrated safety and the ability to elicit immune responses when combined with ICIs [144]. Although loss-of-function mutations are limited in directly exposing antigens on cancer cells, vaccines targeting associated overexpressed proteins hold potential for effectively suppressing tumors.

## 5. Conclusions

In CRC, signature gene mutations play a pivotal role in tumor growth, progression, and the formation of the tumor microenvironment, highlighting their potential as targets for cancer vaccines. Current clinical trials and research efforts in cancer vaccine development have primarily focused on *KRAS* and *BRAF* mutations due to their ability to generate strong neoantigens. However, tumor heterogeneity and complex immune evasion mechanisms complicate therapeutic strategies, and the need for personalized approaches tailored to individual tumor characteristics presents a major limitation. Nonetheless, successful applications of cancer vaccines in other solid tumors offer promise for colorectal cancer treatment. For instance, neoantigen-based approaches and combination strategies with ICIs—proven effective in melanoma and lung cancer—may also be applied to colorectal cancer, a solid tumor, to develop effective therapeutic strategies [158,159]. Personalized approaches using individualized cancer vaccines, improvements in neoantigen prediction technologies, and advances in diverse vaccine platforms collectively support the potential for developing effective cancer vaccines for colorectal cancer. Notably, emerging technologies such as mRNA-based vaccines may facilitate the development of personalized vaccines targeting colorectal cancer-specific antigens [52,160]. In parallel, the development of advanced bioinformatics tools has played a crucial role in supporting personalized vaccine strategies. Platforms such as NetMHCpan and pVACtools assist in predicting immunogenic neoantigens and optimizing HLA typing, thereby streamlining the design of individualized mRNA-based vaccines [161,162]. As these computational approaches continue to advance, they are expected to narrow the gap between molecular tumor profiling and clinical application, ultimately making the translation of personalized immunotherapies into clinical practice. Future research should prioritize integrated strategies targeting multiple signature genes simultaneously. Additionally, elucidating the synergistic effects of combining cancer vaccines with conventional therapies is essential. In conclusion, cancer vaccines hold the potential to redefine colorectal cancer treatment and, with continued research, may offer more effective and personalized therapeutic options for patients.

## Figures and Tables

**Table 1:** Cancer vaccines targeting signature genes in CRC.

Drug/Formulation	Related Signature Genes	Mechanism	Clinical Trial Phase	Combination/ Adjuvant	Reference
GVAX/Cell-based	KRAS	Secretes GM-CSF to activate DC	II	Pembrolizumab	[134]
ELI-002/Lipopeptide-based	KRAS	AMP-CpG activates immune system, AMP-mKRAS peptide targets KRAS	Ib	-	[114,142]
-/Long peptide-based	KRAS	Increases diversity of T-cell responses by long peptide	I	Nivolumab, Ipilimumab	[116]
TG01/Peptide-based	KRAS	Enhances DTH response and T cell proliferation	I/II	QS-21	[135]
E-39/Peptide-based	NRAS/KRAS	TALs from E39-stimulated induce cytolysis in tumor	I/II	-	[137]
KISIMA-Ascl2 vaccine/Peptide-based	APC	Utilizing Ascl2 upregulated in ISCs with KISIMA vaccine platform	-	Anti-PD-1/AS15	[138]
BNT122/mRNA-based	KRAS/BRAF	Upregulates tumor-specific cytotoxic immune response	II	Atezolizumab	[139]
mRNA5681(V941)/ mRNA-based	KRAS	Administered mRNA induces antigen expression	II	Pembrolizumab	[140]
GRT-R904/saRNA-based, Vector-based	KRAS	Prime-boost strategy with ChAdV and saRNA-LNP combined	I/II	Keytruda, Pembrolizumab	[133,143]
Pexa-Vec(JX-594)/ Vector-based	KRAS/TP53	Oncolytic virus-based that directly destroys tumors and promotes immune response	I/II	-	[131,132]
Nous-209/Vector-based	MMR	Prime-boost strategy with GAd20-209-FSP eliciting robust T cell response	Ib/II	Pembrolizumab	[141,144]

DC, dendritic cell; GM-CSF, granulocyte-macrophage colony-stimulating factor; DTH, delayed-type hypersensitivity; TAL, tumor-associated lymphocyte; ISC, intestinal stem cell; saRNA, self-amplifying RNA; ChAdV, chimpanzee adenovirus; GAd, gorilla adenovirus; FSP, frameshift peptide.

## Abbreviations

The following abbreviations are used in this manuscript:

AKT	Protein kinase B
APC	Adenomatous polyposis coli
APCs	Antigens-presenting cells
Ascl2	Achaete-scute family bHLH transcription factor 2
BRAF	B-Raf proto-oncogene, serine/threonine kinase
CAFs	Cancer-associated fibroblasts
ChAd	Chimpanzee adenovirus
CK Iα	Casein kinase I alpha
CRC	Colorectal cancer
CTLs	Cytotoxic T lymphocytes
dMMR	Mismatch repair deficiency
DC	Dendritic cell
DTH	Delayed-type hypersensitivity
ECM	Extracellular matrix
EPCAM	Epithelial cell adhesion molecule
ERK	Extracellular signal-regulated kinase
FSP	Frameshift peptide
GaD	Gorilla adenovirus
GM-CSF	Granulocyte-macrophage colony-stimulating factor
GSK-3β	Glycogen synthase kinase-3β
HIF-1α	Hypoxia-inducible factor-1 alpha
HLA	Human leukocyte antigen
ICIs	Immune checkpoint inhibitors
iCAFs	Inflammatory CAFs
ISC	Intestinal stem cell
KRAS	Kirsten rat sarcoma virus
LNP	Lipid nanoparticle
MEK	Mitogen-activated protein kinase
MHC	Major histocompatibility complex
MKI67	Marker of proliferation Ki-67
MLH1	mutL homolog1
MMR	Mismatch repair
MDSCs	Myeloid-derived suppressor cells
MSI	Microsatellite instability
MSI-H	Microsatellite instability-high
MSS	Microsatellite stable
mTORC1	Mammalian target of rapamycin complex1
myoCAFs	Myofibroblastic CAFs
MSH2	muts homolog2
MSH6	muts homolog6
NO	Nitric oxide
pDNA	Plasmid DNA
PD-L1	Programmed cell death-ligand 1
PCD	Programmed cell death
PI3K	Phosphatidylinositol-3-kinase
PMS2	Postmeiotic segregation increased2
PCD	Programmed cell death
ROS	Reactive oxygen species
samRNA	Self-amplifying mRNA
SLP	Synthetic long peptide
TAL	Tumor-associated lymphocyte
TAMs	Tumor-associated macrophages
TAAs	Tumor-associated antigens
TILs	Tumor-infiltrating lymphocytes
TMB	Tumor mutation burden
TME	Tumor microenvironment
Treg	Regulatory T cell
TSAs	Tumor-specific antigens
